# Discriminative Localized Sparse Approximations for Mass Characterization in Mammograms

**DOI:** 10.3389/fonc.2021.725320

**Published:** 2021-12-30

**Authors:** Sokratis Makrogiannis, Keni Zheng, Chelsea Harris

**Affiliations:** Math Imaging and Visual Computing Lab, Division of Physics, Engineering, Mathematics and Computer Science, Delaware State University, Dover, DE, United States

**Keywords:** computer-aided diagnosis (CADx), sparse approximation, breast cancer screening, mass classification, mammographic imaging

## Abstract

The most common form of cancer among women in both developed and developing countries is breast cancer. The early detection and diagnosis of this disease is significant because it may reduce the number of deaths caused by breast cancer and improve the quality of life of those effected. Computer-aided detection (CADe) and computer-aided diagnosis (CADx) methods have shown promise in recent years for aiding in the human expert reading analysis and improving the accuracy and reproducibility of pathology results. One significant application of CADe and CADx is for breast cancer screening using mammograms. In image processing and machine learning research, relevant results have been produced by sparse analysis methods to represent and recognize imaging patterns. However, application of sparse analysis techniques to the biomedical field is challenging, as the objects of interest may be obscured because of contrast limitations or background tissues, and their appearance may change because of anatomical variability. We introduce methods for label-specific and label-consistent dictionary learning to improve the separation of benign breast masses from malignant breast masses in mammograms. We integrated these approaches into our Spatially Localized Ensemble Sparse Analysis (SLESA) methodology. We performed 10- and 30-fold cross validation (CV) experiments on multiple mammography datasets to measure the classification performance of our methodology and compared it to deep learning models and conventional sparse representation. Results from these experiments show the potential of this methodology for separation of malignant from benign masses as a part of a breast cancer screening workflow.

## 1 Introduction

The topic of this work is automated classification of breast masses into benign or malignant using mammograms. The diagnosis of breast cancer is an impactful domain of research (1), therefore, automated methods of detection and diagnosis of breast cancer have gained popularity in the past few decades (2–6). Early diagnosis of breast cancer has been shown to reduce mortality related to this disease and significantly improve the quality of life of those affected. To achieve early diagnosis, mammograms are used to aid in detecting breast cancer. Proper detection and diagnosis of breast abnormalities requires the experience and high levels of expertise of trained radiologists. Computer-aided diagnosis would improve the reproducibility of diagnosis states and reduce the time spent to thoroughly diagnosis breast cancer.

The X-ray mammographic test is a commonly used method for early prediction and diagnosis of breast cancer (7). Therefore, the development of CADe and CADx techniques for breast cancer using mammograms has attracted significant interest. Among these techniques, conventional classification models use specific procedures to craft features for representing and classifying imaging pattern. Such conventional approaches are introduced in (8–13). Features such as shape, texture, and intensity were extracted in (9). Among the extracted features, the genetic algorithm (GA) selected the most relevant features. Additionally, feature extraction through Zernike moments have been used because of their useful ability to well describe shape characteristics (14). In recent years, feature extraction and selection has been achieved through state-of-the-art techniques that use neural networks (NN) (15). A popular group of NN techniques use Convolutional Neural Nets (CNNs) for classification. Key advances in both the design and application of CNNs (16, 17) led to significant improvement in the state-of-the-art object recognition on the Imagenet dataset. A common training method used for CNNs is transfer learning; this technique has been applied to medical imaging for classification tasks (15, 18, 19). In (20), for example, pretrained VGG16, ResNet50, and Inception v3 networks were customized and applied to several mammographic datasets.

The concentration of this research is the diagnosis (CADx) of breast cancer masses into benign or malignant states using sparse representation and dictionary learning techniques. Sparse representation has been applied in the areas of computer vision, signal/image processing, and pattern recognition. The objective of sparse representation methods is to use sparse linear approximations of patterns, or atoms, from a dictionary of signals to represent a specific signal. These sparse approximations can then be used for applications such as compression and denoising of signals/images, classification, object recognition, and other areas. A common area of interest in such techniques is dictionary learning. Dictionary learning focuses on the methods for learning dictionaries in order to obtain optimal representations according to the application objective. Dictionary learning techniques have produced impressive results in a variety of signal and image processing applications (21–30). In more recent years, a widely studied area has been convolutional sparse coding, and its relationship with deep learning techniques (27, 30, 31).

Although there is substantial interest in the aforementioned techniques, their application to the biomedical field remains within limits to the straightforward utilization of sparse representation classification (SRC), or learning of multiple separate dictionaries. Hence motivation remains for the design of methods that leverage the capabilities of dictionary learning and sparse coding using joint discriminative-generative approaches.

Here we propose the integration of discriminative dictionary learning methods into our spatially localized ensemble sparse analysis classification (SLESA) model. Our dictionary learning techniques incorporate class label separation and label consistency and we denote these variations as LS-SLESA and LC-SLESA respectively. We train multiple dictionaries on the same set of ROIs and fuse the residuals of multiple approximations to obtain more robust class estimates than those obtained by single dictionary learning as also supported by (32). Our premise is that optimized spatially localized dictionaries trained using label separation or label consistency constraints, will improve the classification accuracy of our spatially localized sparse analysis. We employ this system for diagnosis of breast cancer in mammograms. We evaluate the performance of our framework and compare it to straightforward sparse representation classification (SRC), and the well-known CNN architectures of Alexnet (16), Googlenet (17), Resnet50 (33), and InceptionV3 (34), after applying transfer learning and data augmentation techniques.

### 1.1 Sparse Analysis

In recent years, the research area of sparse representation of signals has attracted considerable interest. The central focus of sparse analysis is to optimize an objective function. The objective function is comprised of a reconstruction error term and a sparsity term. The reconstruction error term or the residual, produces the measurement of the difference between the signal reconstruction and the test signal. The sparsity term measures the sparsity of the computed solution. The residual term may be set to measure the test signal exactly or within a defined bound of constraint.

In image classification tasks, the sparse representation of a test image is used to assign that image to a class. Sparse representation-based classification has two phases: coding and classification. In the coding phase, an image or signal is collaboratively coded with a dictionary of atoms given a sparsity constraint. The classification of the image is performed based on the coding coefficients and the dictionary. One of the advantages of sparse representation in image classification tasks is its ability to represent a high-dimensional image by few representative samples.

The dictionary *D* consists of columns of signals, also called atoms. The design of the dictionary could be simply predefined. For example, a dictionary that consists of all training samples from all classes is considered predefined. However, dictionaries of this form may fail to represent test samples well, if the atoms are inter-correlated, or they do not span the range of the image content. Moreover, very large dictionaries increase the coding complexity.

Sparse analysis solves the following optimization problem: given signals in an ℝ*^d^* space, a dictionary *D* ∈ ℝ*^d^* ^×^ *^n^* of signals partitioned by class, and a test signal *y* ∈ℝ*^d^*, sparse coding seeks to find a coding vector $\hat{x}$ ∈ℝ*^n^*. The test signal *y* is represented as a linear combination of the dictionary atoms and a sparse code. This mathematical optimization problem is expressed by


(1) $$\hat{x}=\arg\underset{x}{\min}||\hat{x}|{|}_{0}\;subject\;to\;y=Dx.$$

Sparsity is represented by the *ℓ*_0_ norm, but may also be approximated by the *ℓ*_1_ norm, or *ℓ_p_* norms where *p* ∈(0,1). Assuming that the signal contains noise, we can introduce ∈ as a tolerance parameter and solve the following problem,


(2) $$\hat{x}=\arg\underset{x}{\min}||\hat{x}|{|}_{0}\;subject\;to\;||y-Dx||<\varepsilon$$

Pursuit algorithms such as basis pursuit (BP) and orthogonal matching pursuit (OMP) are often used to solve the sparse coding problems defined in Equations 1 and 2. Basis pursuit is a linear programming technique that seeks to find the sparsest *L*_1_ solution to to the mathematical optimization problem defined in Equation 1. The orthogonal matching pursuit is considered a greedy pursuit algorithm in that it updates the sparse solution vector coefficients using previously updated solution vector atoms. OMP is a more complex and computationally expensive extension of the matching pursuit algorithm (MP), however, can often lead to better sparse solutions.

Early sparse representation techniques such as SRC (35), optimize an objective function of two terms, and design the dictionary *D* with the original training images as dictionary columns or atoms. In more recent works, we see an emphasize on the design of the dictionary and task-specific optimization, of which we discuss in the next section.

### 1.2 Dictionary Learning

As discussed before, the dictionary is a key component of the optimization problem. Learning a dictionary from training data has been an area of interest in recent years (25, 36). The goal of such techniques is to construct dictionaries optimized for class representation and separation. Previous works have shown that dictionary learning may improve the performance of image processing and recognition tasks (25). Dictionary learning techniques can be divided into the following groups (23): (i) probabilistic learning methods, (ii) clustering-based learning methods, and (iii) construction methods.

The type, design, and dimensions of the dictionary have a significant effect on the solutions of the sparse optimization problem. The atoms are expected to be able to approximate the variations of the specific image domain and have low correlation with each other. Considering the dictionary dimensions, a dictionary is considered overcomplete when the number of signals within the dictionary (*n*) exceeds the dimension of the signal to be represented (*d*), that is if *d*<*n*. Overcomplete dictionaries are required to produce sparse representations of signals (37).

## 2 Methodology

**Figure 1:**
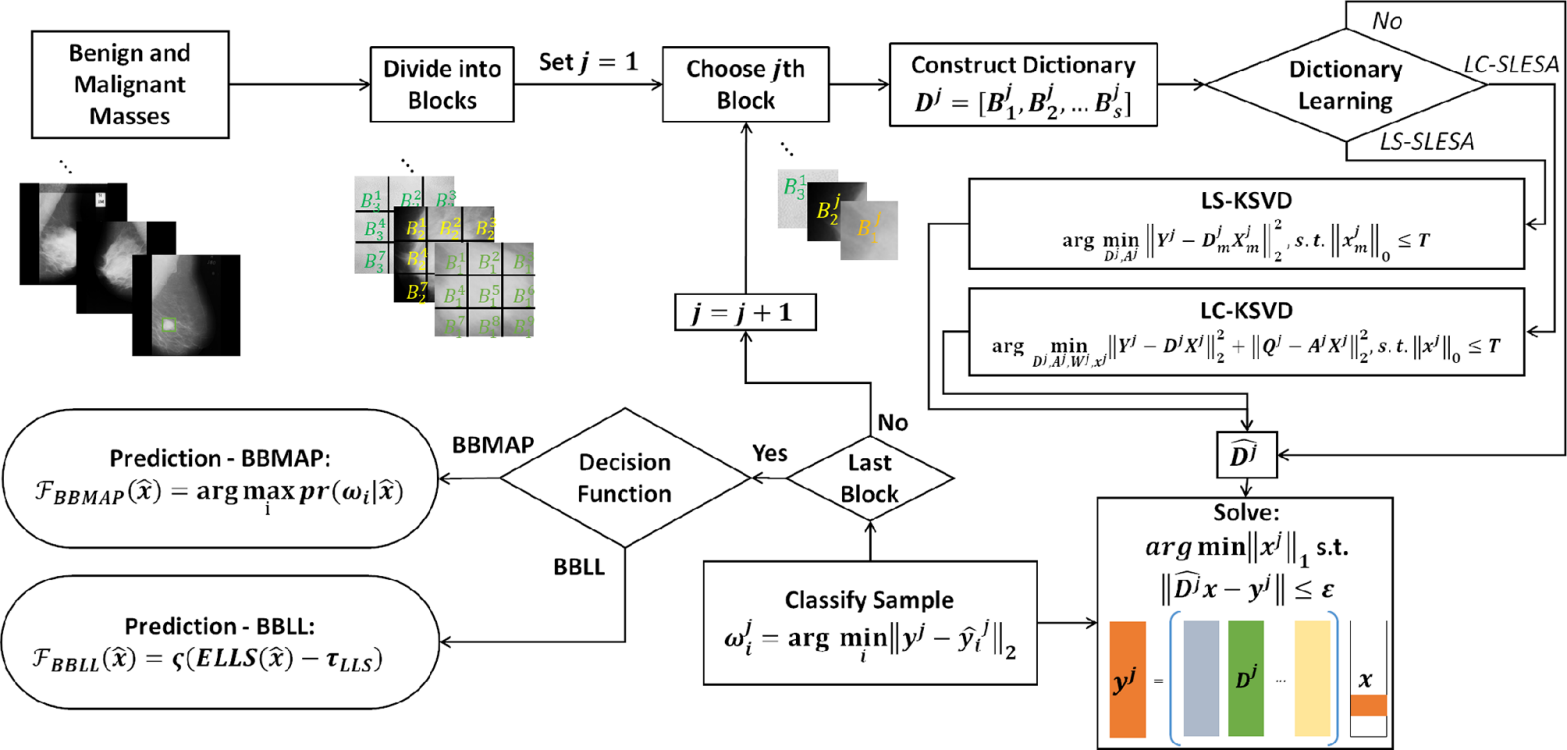
Main stages of the proposed methodology; block decomposition, dictionary learning, and ensemble classification. ROIs of benign and malignant image samples are first divided into blocks. Block dictionaries are constructed for each block index overall the training samples. Each block of a test image is classified using the corresponding block dictionary. If no dictionary learning is performed, our SLESA method is employed and an image is classified using an ensemble of its block classifications. When dictionary learning is used, either through KSVD (our LS-SLESA method) or through LC-KSVD2 (our LC-SLESA method) the block dictionaries are learned to produce more discriminative dictionaries. Individual spatially localized decisions are combined to classify test samples using ensemble techniques BBMAP and BBLL-S or -R.

In this work, we introduce class label separation and class label consistency into the localized dictionaries within our spatially localized sparse analysis (SLESA) framework. We denote the respective methods by LS-SLESA and LC-SLESA. Our SLESA approach applies localized block decomposition that reduces the length of the feature vector and helps to build overcomplete dictionaries. In the classification stage, we solve the sparse representation problem for each block using orthogonal matching pursuit (OMP), and fuse the individual block-wise responses to determine the lesion category. LS-SLESA and LC-SLESA aim to further improve the performance of our previous work, SLESA, by finding task-specific dictionaries that utilize the class labels of the training data. We consider two approaches: one calculates separate dictionaries for benign and malignant breast masses, and the other incorporates linear classification errors into the optimization problem. **Figure 1** outlines the main stages of our methodology.

### 2.1 Spatially Localized Block Decomposition

We divide each training image *I* into *m*×*n* px blocks that are spatially ordered. Therefore, *I* = [B^1^, B^2^, …, B*^NB^*], where *B^j^* denotes a block of each training image and *NB* is the total number blocks of an image. We construct dictionaries *D^j^*, where *j* = 1,2,…, *NB*, from the same position of the block *B^j^* for all *s* images of the training set:


(3) $$D^{j}=[B_{1}^{j},B_{2}^{j},\cdots B_{s}^{j}].$$

Therefore, a number of *NB* block dictionaries are constructed, each unique in the spatial information that they provide to classify spatially localized image blocks.

### 2.2 Label Specific Spatially Localized Ensemble Sparse Analysis

We introduce dictionary learning techniques to improve the sparse approximation accuracy and generalizability. We learn a separate dictionary for each type of mass and we then merge the dictionaries to perform sparse coding and classification.

We employ the KSVD algorithm by (21) to learn the dictionary. KSVD updates the atoms of the dictionary by iteratively solving sparse coding problems that alternate between residual and sparsity constraints. The optimized atom in each iteration is computed by Singular Value Decomposition (SVD). This method has been shown to converge to effective solutions and has been widely applied for sparse representation.

After the block decomposition step, we learn *NB* discriminative dictionaries using block-based label-separated KSVD. We denote this approach by LS-SLESA.


(4) $$\arg\underset{D^{j},A^{j}}{\min}||Y^{j}-D_{m}^{j}X_{m}^{j}|{|}_{2}^{2}\;s.t.\;||x_{m}^{j}|{|}_{0}\leq T,$$

where *Y^j^* denotes the training block samples. We solve the above problem for each class index *m*, and then concatenate the class-specific dictionaries $D_{m}^{j}$ to form the complete dictionary *D^j^* for the *j*-th block.

### 2.3 Label Consistent Spatially Localized Ensemble Sparse Analysis

Another approach is to learn *NB* discriminative dictionaries using the label consistent KSVD algorithm (denoted by LC-KSVD). Sparse coding and sparse classification errors are added to the optimization problem in order to compute a single discriminative dictionary. We employ LC-KSVD to learn the dictionaries *D^j^*. The authors in (24) proposed two variants named LC-KSVD1 and LC-KSVD2. In their work, classification performance was consistently greater when the LC-KSVD2 variant is used versus the LC-KSVD1 dictionary learning approach. Thus, we employed the objective function of LC-KSVD2 in our LC-SLESA approach. Thus, omitting the need for ablation experiments on the effectiveness of the loss terms in the LC-KSVD methods.

LC-KSVD2 adds a label consistency regularization term and a joint classification error term to the objective function. The optimization problem is:


(5) $$\begin{array}{l} \text{arg }\underset{D^{j},A^{j},W^{j},x^{j}}{\text{min}}||Y^{j}-D^{j}X^{j}|{|}_{2}^{2}+||Q^{j}-A^{j}X^{j}|{|}_{2}^{2}+\;||H^{j}-W^{j}X^{j}|{|}_{2}^{2}\\ s.t.\;||x^{j}|{|}_{0}\leq T. \end{array}$$

*Q^j^* denotes the class-specific sparse codes for *Y^j^*, and *A^j^* is a linear transformation matrix. *W^j^* symbolizes the parameters of the linear classifier, and *H^j^* contains the class labels of the training data *Y^j^*. *T* is the sparsity threshold. The term $||Q^{j}-A^{j}X^{j}|{|}_{2}^{2}$ is the discriminative sparse code error that forces patterns from the same class to have similar sparse codes. *Q^j^* is defined as $Q^{j}=[q_{1}^{j},\ldots ,q_{N}^{j}]$ for *N* many training samples where the discriminative sparse codes for a sample, $q_{i}^{j}$ contains zero indices where the training sample $y_{i}^{j}\in Y^{j}$ and its corresponding dictionary do not share the same class label. The term $||H^{j}-W^{j}X^{j}|{|}_{2}^{2}$ expresses the classification error.

### 2.4 Ensemble Classification

In this stage of our method, we combine the individual spatially localized decisions to classify the test samples. We find the solution *x^j^* of the regularized noisy *ℓ*_1_-minimization problem, for each test sample *y^j^* corresponding to the *j*th block:


(6) $${\hat{x}}^{j}=\arg\min||x^{j}|{|}_{1}\;subject\;to\;||D^{j}x-y^{j}|{|}_{2}\leq \;\varepsilon$$

We propose ensemble learning techniques in a Bayesian probabilistic setting to fuse classifier predictions. We propose a decision function that applies majority voting to individual hypotheses (BBMAP), and an ensemble of log-likelihood scores (BBLL) computed from either the sparsity of the solution (BBLL-S), or approximation residual (BBLL-R).

#### 2.4.1 Maximum a Posteriori Decision Function (BBMAP)

The class label of a test sample is determined by the MAP estimate produced by *NB* block-based classifiers. The predicted class label $\hat{\omega }$ is


(7) $${\hat{\omega }}_{BBMAP}=F_{BBMAP}(\hat{x})≐\arg\underset{i}{\max}\;pr(\omega_{i}|\hat{x}),$$

where $pr(\omega_{i}|\hat{x})$ is the posterior probability for class *ω_i_* given $\hat{x}$ .

#### 2.4.2 Log Likelihood Sparsity-Based Decision Function (BBLL-S)

This decision function first computes a log-likelihood score based on the relative sparsity scores $||\delta_{m}({\hat{x}}^{j})|{|}_{1},\;||\delta_{n}({\hat{x}}^{j})|{|}_{1}$ , obtained from the sparse representation stage of each classifier


(8) $$LLS({\hat{x}}^{j})=-\text{log }\frac{{\left\|\delta_{m}({\hat{x}}^{j})\right\|}_{1}}{{\left\|\delta_{n}({\hat{x}}^{j})\right\|}_{1}}\;\{\begin{array}{c} \begin{array}{cc} \geq 0, & {\hat{x}}^{j} \end{array}\in \;m\text{th class}\\ \begin{array}{cc} <0, & {\hat{x}}^{j} \end{array}\in n\text{th class} \end{array}$$

We estimate the expectation of $LLS^{j}(\hat{x})$ that we denote by *ELLS* over the individual classification scores obtained by (8)


(9) $$\begin{array}{l} ELLS(\hat{x})\overset{\;}{≐}E\{LLS({\hat{x}}^{j})\}=\frac{1}{NB}\overset{NB}{\underset{j}{\Sigma }}LLS({\hat{x}}^{j})\\ =-\frac{1}{NB}\left[\overset{NB}{\underset{j}{\Sigma }}\log{\left\|\delta_{m}({\hat{x}}^{j})\right\|}_{1}-\overset{NB}{\underset{j}{\Sigma }}\log{\left\|\delta_{n}({\hat{x}}^{j})\right\|}_{1}\right]. \end{array}$$

We apply a sigmoid function *ς*(.)to produce classification scores in the range of [–1,1]. We employ a shift parameter τ*_LLS_* to account for classification bias,


(10) $$F_{LLS}(\hat{x})≐\varsigma (ELLS(\hat{x})-\tau_{LLS}).$$

The final decision is given by the sign of $F_{LLS}(\hat{x})$ :


(11) $${\hat{\omega }}_{LLS}(\hat{x})=Sgn\{F_{LLS}(\hat{x})\}.$$

#### 2.4.3 Log Likelihood Residual-Based Decision Function (BBLL-R)

This function computes a log-likelihood score based on the relative residual scores $||\delta_{m}({\hat{x}}^{j})|{|}_{1},\;||\delta_{n}({\hat{x}}^{j})|{|}_{1}$ , obtained from the sparse representation stage,


(12) $$LLR({\hat{x}}^{j})=-log\;\frac{{\left\|D^{j}\delta_{m}({\hat{x}}^{j})-y^{j}\right\|}_{2}}{{\left\|D^{j}\delta_{n}({\hat{x}}^{j})-y^{j}\right\|}_{2}}\;\{\begin{array}{c} \begin{array}{cc} \geq 0, & {\hat{x}}^{j} \end{array}\in \;m\text{th class}\\ \begin{array}{cc} <0, & {\hat{x}}^{j} \end{array}\in n\text{th class} \end{array}$$

We estimate the expectation of $LLR(\hat{x})$ , denoted by *ELLR*, over all the individual classification scores obtained by (12),


(13) $$ELLR(\hat{x})≐E\{LLR({\hat{x}}^{j})\}=\frac{1}{NB}\sum_{j}^{NB}LLR({\hat{x}}^{j})$$

We apply a sigmoid function *ς*(.)with a shift parameter *τ_LLR_* and a sign function, to determine the state of $\hat{x}$ , symbolized by ${\hat{\omega }}_{LLR}(\hat{x})$ , as in (10, 11).

## 3 Experiments and Discussion

We evaluated our method for classification of breast masses into malignant or benign states on two digital mammographic databases. Next, we describe our experiments and report results produced by our approach. For comparison, we report the results of variants to our proposed method including straightforward sparse representation and multiple strategies for dictionary learning in SLESA, LS-SLESA and LC-SLESA. These may serve as ablation experiments to evaluate the effect of ensemble classification and the effect of dictionary learning on the performance of our method. We have also validated the performance of widely used convolutional neural networks (16, 17, 33, 34), after applying transfer learning, random resampling, and extensive optimization.

### 3.1 Datasets

The training and testing data used in our experimentation were obtained from the Mammographic Image Analysis Society (MIAS) (2) and the Digital Database for Screening Mammography (DDSM). The Mammographic Image Analysis Society (MIAS) database is one of oldest and the most widely used mammography databases. The resolution of the mammograms is 200-micron pixel edge that is approximately equivalent to 264.58 *μm* pixel size. The image size after clipping or padding is 1024×1024 px. The MIAS dataset consists of 322 digitized mediolateral oblique (MLO) images (68 benign, 51 malignant, 203 normal). We selected mammograms containing 51 malignant and 66 benign masses in total, to evaluate classification performance. The Digital Database for Screening Mammography (DDSM) is a large public database including a total of 10,480 images. CBIS-DDSM (Curated Breast Imaging Subset of DDSM) is a carefully selected and updated subset DDSM (Digital Database in for Screening Mammography). It contains 753 calcification subjects and 891 mass subjects. In our experiments we used the CC view (craniocaudal view) of benign and malignant lesions of CBIS-DDSM (Curated Breast Imaging Subset of DDSM). Thus, the number of malignant cases used in our experiments was narrowed down to 296 malignant and 311 benign cases.

To prepare the data for the first stage of our method, block decomposition, we first selected regions of interest (ROIs) containing the masses. Our method reads-in two key values from radiological readings, that is, the centroid and radius of each mass. It determines a minimum bounding square ROI and select the masses that satisfy a size criterion. In the first approach, we ensured that the majority of the blocks cover the complete mass area. The mass ROI sizes are required to be greater than, or equal to a fixed ROI size. The qualifying masses are center-cropped to generate the ROI data. In the second approach, we selected the complete ROIs including background tissue using the mass centroid and radius. Then we resampled all ROIs to a fixed size, instead of applying a minimum size criterion. In MIAS data we followed both approaches for ROI selection. In the CBIS-DDSM data we followed the second approach. We performed 10- and 30-fold cross-validation on the ROIs to examine the effect of the cross-validation fold size on performance.

### 3.2 Convolutional Neural Networks With Transfer Learning

For comparison purposes, we implemented CNN classifiers using the Alexnet (16), Googlenet (17), Resnet50 (33), and InceptionV3 (34) architectures with transfer learning. All networks were pre-trained on the Imagenet database that contains 1.2 million natural images.

Transfer learning was applied to each network in various ways. To modify Alexnet to our data, we replaced the pre-trained fully connected layers with three new fully connected layers. The learning rates of the pre-trained layers were set to 0 in order to keep the network weights fixed. We only trained the new fully connected layers. For Googlenet, the learning rates of the bottom 10 layers were set to 0, and the top fully connected layer was replaced with a new fully connected layer. We also assigned a greater learning rate factor for the new layer than the pre-trained layers. In Resnet50, we replaced the pre-trained fully connected layers with three new fully connected layers. We set the learning rates of the pre-trained layers to 0, in order to train only the new fully connected layers. In InceptionV3, we replaced the top classification layers with three new fully connected layers. We set the learning rates of the pre-trained layers of InceptionV3 to 0, as we did in Alexnet and Resnet50.

To provide the networks with additional training examples, we applied data resampling using randomly-centered patches inside each ROI. Additionally, we applied data augmentation by rotation, scaling, and horizontal and vertical flipping. Finally, we used Bayesian optimization (38, 39) to tune the learning rate, mini-batch size, and number of epochs.

Due to the ability of deep networks to learn information from the edges of masses and not just the texture, we decided to test our method on 256×256 px ROIs of all masses including the background tissue in the MIAS database (66 benign and 51 malignant). **Table 1** summarizes the results of our cross-validation experiments. Googlenet yields the top ACC of 67.65% and the top AUC of 63.04% for 30-fold cross-validation.

**Table 1:** Breast mass classification performance on MIAS data using convolutional neural network classifiers (ROI size: **256 × 256**).

Method	k-Fold CV	ROI Size	TPR (%)	TNR (%)	ACC (%)	AUC (%)
Alexnet	10	256 × 256	56.86	72.55	**64.71**	**62.19**
	30	256 × 256	58.82	64.71	61.77	60.29
Googlenet	10	256 × 256	64.71	58.82	61.77	57.86
	30	256 × 256	66.67	68.63	**67.65**	**63.04**
Resnet50	10	256 × 256	60.78	62.75	61.76	57.32
	30	256 × 256	44.12	55.88	53.6	56.8
InceptionV3	10	256 × 256	58.82	60.78	59.80	58.59
	30	256 × 256	58.82	60.78	59.80	57.44

The top performances of 10- and 30-fold cross-validation are shown in bold.

When using DDSM data, we applied the same ROI selection strategy with that of MIAS. The Alexnet architecture yields the top ACC of 69.59% and the top AUC of 73.04% using 30-fold cross-validation (**Table 2**). We note the increase in classification performance when using DDSM for training and testing. This is expected, because CNNs require a large number of diverse training samples to achieve good performance. DDSM is a larger database than MIAS, therefore CNNs are able to learn more relevant features for classification. Of note is that simpler networks such as Alexnet and Googlenet, with smaller numbers of trainable weights, produce more accurate classifications than deeper networks such as InceptionV3. This is expected because of the limited number of training samples in both datasets.

**Table 2:** Breast mass classification performance on DDSM data using convolutional neural network classifiers (ROI size: **256 × 256**).

Method	k-Fold CV	ROI Size	TPR (%)	TNR (%)	ACC (%)	AUC (%)
Alexnet	10	256 × 256	67.57	65.88	**66.72**	**69.70**
	30	256 × 256	72.64	66.55	**69.59**	**73.04**
Googlenet	10	256 × 256	72.64	59.46	66.05	69.55
	30	256 × 256	66.89	64.19	65.5	69.43
Resnet50	10	256 × 256	56.42	75.68	66.05	70.35
	30	256 × 256	60.81	73.31	67.06	71.34
InceptionV3	10	256 × 256	61.82	67.57	64.70	64.70
	30	256 × 256	65.20	64.19	64.70	66.94

The top performances of 10- and 30-fold cross-validation are shown in bold.

### 3.3 LS-SLESA and LC-SLESA

**Figure 2:**
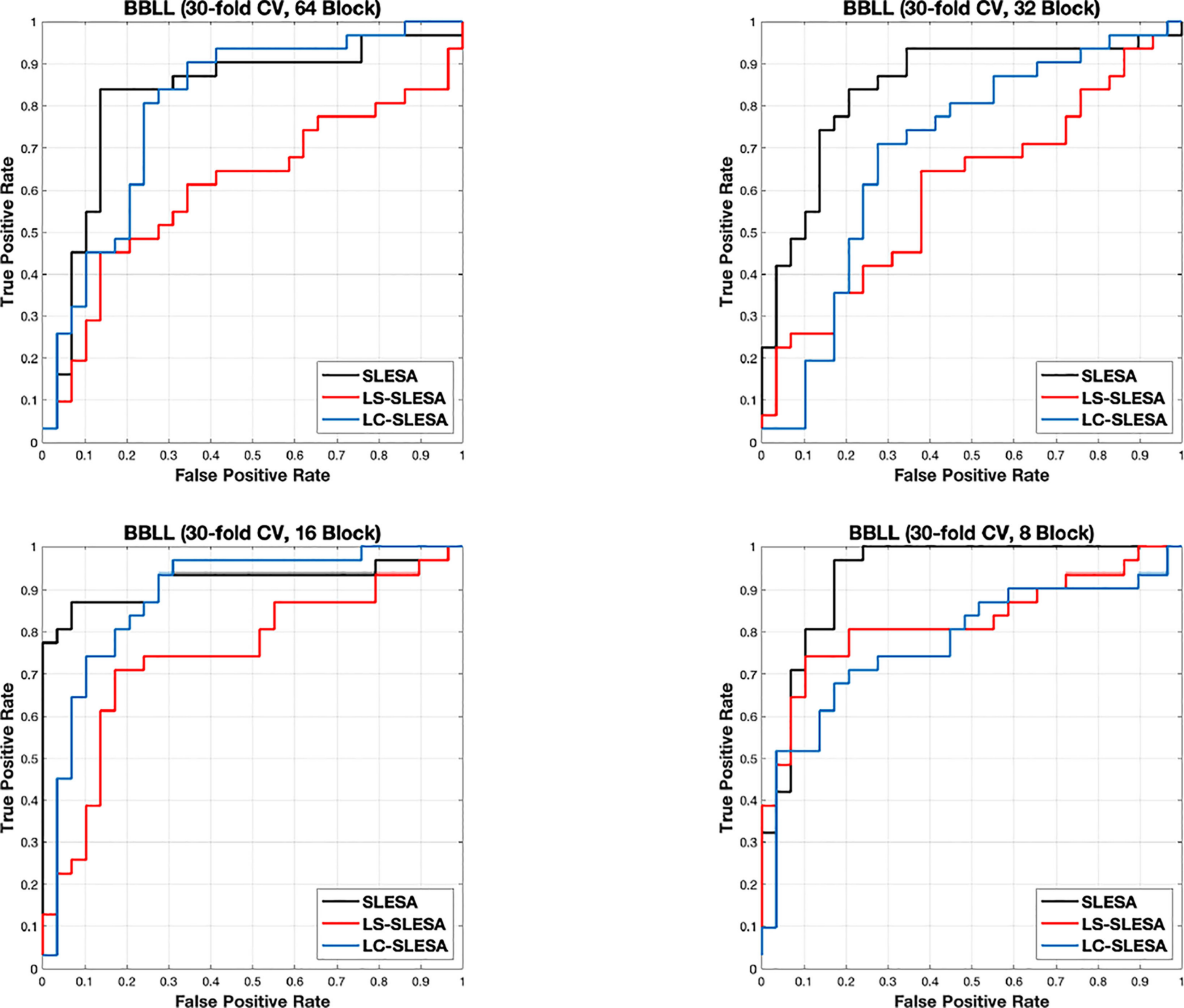
ROC plots for **64** × **64**, **32** × **32**, **16** × **16**, and **8** × **8** block sizes using the proposed block-based ensemble method on the MIAS dataset with BBLL decision functions and 30-fold CV.

Next, we evaluated the performance of our block-based ensemble classification method by 10- and 30-fold cross-validation. In the MIAS section of our experiments, we present results using minimum ROI size of 64×64 pixels, resulting in a dataset of 36 benign and 37 malignant masses. In **Table 3**, we report the classification rates produced for multiple block sizes. When the block size is equal to the ROI size, conventional SRC is performed (35); these results are reported in the first row of **Table 3**. We observe that ACC and AUC generally increase when the number of folds increases, for the same ROI size. The top ACC using 10-fold cross-validation is 72.86% for 8×8 block size by SLESA, and for 64×64 block size by LS-SLESA with BBLL-S decision function. The top AUC for 10-fold CV is 75.35% for 8×8 block size, produced by LS-SLESA. The best overall performance is obtained for 30-fold cross validation. The top accuracy is 90% for 16×16 and 8×8 block sizes by SLESA, and the largest area under the curve is 93.10% for 8×8 block size by SLESA with BBLL-S decision function. In 30-fold cross-validation, 2 or 3 images are tested in each fold. Additionally, in **Table 3** we report true positive rates (TPR) and true negative rates (TNR) for each experiment. Generally, we observe higher true negative rates on average than true positive rates, which is an indication that the positive class, malignant, is more difficult to classify. **Figure 2** displays the receiver operating curves (ROC) by SLESA, LS-SLESA and LC-SLESA for 64,32,16 and 8px block lengths using 30-fold CV. The ROC graphs are consistent with the results in **Table 3**. We compare BBLL-S ROC curves in **Figure 2** among the SLESA methods by applying DeLong’s statistical test for 30-fold cross-validation on the MIAS dataset. These tests produced statistically significant differences in AUCs at the level *α* = 0.05 between SLESA and LS-SLESA for 64,32, and 16px block lengths. These tests determined as significant, AUC differences between SLESA and LC-SLESA for 8px block length, and between LS-SLESA and LC-SLESA for 64px block length. The results indicate that SLESA produced better AUC values in 30-fold CV.

**Table 3:** Breast mass classification performance on MIAS data using ensembles of block-based sparse classifiers with dictionary learning (ROI size: **64×64**).

Method	k-Fold	Block Size	SLESA	SLESA	SLESA	SLESA	LS-SLESA	LS-SLESA	LS-SLESA	LS-SLESA	LC-SLESA	LC-SLESA	LC-SLESA	LC-SLESA
	CV		TPR (%)	TNR (%)	ACC (%)	AUC (%)	TPR (%)	TNR (%)	ACC (%)	AUC (%)	TPR (%)	TNR (%)	ACC (%)	AUC (%)
BBMAP-S	10	64×64	45.95	84.85	64.29	63.55	64.86	81.82	72.86	70.11	75.68	36.36	57.14	53.81
		32×32	51.35	87.88	68.57	69.53	62.16	81.82	71.43	70.84	78.38	36.36	58.57	52.33
		16×16	40.54	90.91	64.29	65.52	59.46	81.82	70.00	69.70	56.76	72.73	64.29	61.26
		8×8	56.76	81.82	68.57	67.90	48.65	81.82	64.29	63.23	62.16	63.64	62.86	60.77
		*Mean*	*48.65*	*86.37*	*66.43*	*66.63*	*58.78*	*81.82*	*69.64*	*68.47*	*68.25*	*52.27*	*60.72*	*57.04*
		*Std Dev*	*6.98*	*3.91*	*2.47*	*2.63*	*7.11*	*0.00*	*3.76*	*3.53*	*10.44*	*18.75*	*3.40*	*4.63*
BBLL-S	10	64×64	64.86	72.73	68.57	70.35	72.97	72.73	**72.86**	71.33	64.86	66.67	65.71	66.42
		32×32	70.27	63.64	67.14	70.02	62.16	81.82	71.43	69.70	70.27	60.61	65.71	68.80
		16×16	59.46	84.85	71.43	**74.37**	59.46	81.82	70.00	69.94	64.86	75.76	**70.00**	**71.42**
		8×8	72.97	72.73	**72.86**	71.58	59.46	81.82	70.00	**75.35**	51.35	81.82	65.71	64.78
		*Mean*	*66.89*	*73.49*	*70.00*	*71.58*	*63.51*	*79.55*	*71.07*	*71.58*	*62.84*	*71.21*	*66.79*	*67.85*
		*Std Dev*	*5.99*	*8.70*	*2.61*	*1.97*	*6.43*	*4.55*	*1.37*	*2.61*	*8.07*	*9.42*	*2.14*	*2.89*
BBMAP-S	30	64×64	22.58	93.10	56.67	52.28	64.52	55.17	60.00	56.62	70.97	62.07	66.67	63.52
		32×32	9.88	100.00	53.33	48.50	48.39	75.86	61.67	59.40	100.00	63.33	63.33	57.17
		16×16	61.29	65.52	63.33	59.96	45.16	82.76	63.33	60.73	75.86	71.67	71.67	69.30
		8×8	38.71	96.55	66.67	61.96	54.84	86.21	70.00	66.07	74.19	55.17	65.00	60.85
		*Mean*	*33.07*	*88.79*	*60.00*	*55.68*	*53.23*	*75.00*	*63.75*	*60.71*	*80.26*	*63.06*	*66.67*	*62.71*
		*Std Dev*	*22.25*	*15.77*	*6.09*	*6.35*	*8.54*	*13.90*	*4.38*	*3.97*	*13.32*	*6.77*	*3.60*	*5.11*
BBLL-S	30	64×64	83.87	86.21	85.00	82.09	45.16	86.21	65.00	60.62	90.32	65.52	78.33	79.98
		32×32	83.87	75.86	80.00	84.43	64.52	62.07	63.33	60.78	61.29	75.86	68.33	69.30
		16×16	87.10	93.10	**90.00**	92.00	70.97	82.76	76.67	74.53	96.77	68.97	**83.33**	**88.43**
		8×8	96.77	82.76	**90.00**	**93.10**	74.19	89.66	**81.67**	**82.43**	67.74	82.76	75.00	77.42
		*Mean*	*87.90*	*84.48*	*86.25*	*87.91*	*63.71*	*80.18*	*71.67*	*69.59*	*79.03*	*73.28*	*76.25*	*78.78*
		*Std Dev*	*6.10*	*7.18*	*4.79*	*5.47*	*13.00*	*12.39*	*8.93*	*10.76*	*17.20*	*7.65*	*6.29*	*7.88*

The top performances of 10- and 30-fold cross-validation are shown in bold.

In the DDSM section of our experiments, we selected the complete ROIs including background tissue using the centroid and radius data. Then we resampled all ROIs to the fixed size of 128×128px. **Table 4** contains a summary of the results. LS-SLESA using 8×8 blocks and BBLL-R decision in 10-fold cross-validation, produces the highest AUC and ACC at 65.34% and 63.17% respectively. Overall, label-specific and label-consistent dictionary learning improves the ACC and AUC.

**Table 4:** Breast mass classification performance on DDSM data using ensembles of block-based sparse classifiers with dictionary learning (ROI size: **128×128**).

Method	k-Fold	Block Size	SLESA	SLESA	SLESA	SLESA	LS-SLESA	LS-SLESA	LS-SLESA	LS-SLESA	LC-SLESA	LC-SLESA	LC-SLESA	LC-SLESA
	CV		TPR (%)	TNR (%)	ACC (%)	AUC (%)	TPR (%)	TNR (%)	ACC (%)	AUC (%)	TPR (%))	TNR (%)	ACC (%)	AUC (%)
BBMAP-R	10	128×128	55.97	49.83	52.83	53.12	69.62	43.65	56.33	56.93	57.00	54.07	55.50	55.82
		64×64	40.61	63.52	52.33	51.90	49.15	64.17	56.83	56.70	48.81	66.45	57.83	57.62
		32×32	54.61	55.70	55.17	55.25	59.04	62.22	60.67	61.00	60.07	54.07	57.00	57.21
		16×16	62.12	50.81	56.33	56.71	75.43	36.81	55.67	55.92	57.68	57.00	57.33	57.49
		8×8	60.07	50.81	55.33	55.84	62.12	56.68	59.33	60.05	51.19	66.45	59.00	58.97
		*Mean*	*54.68*	*54.13*	*54.40*	*54.56*	*63.07*	*52.71*	*57.77*	*58.12*	*54.95*	*59.61*	*57.33*	*57.42*
		*Std Dev*	*8.42*	*5.73*	*1.73*	*1.99*	*10.08*	*11.96*	*2.13*	*2.25*	*4.74*	*6.36*	*1.27*	*1.12*
BBLL-R	10	128×128	44.30	65.87	54.83	53.35	69.97	43.65	56.50	57.17	34.13	77.85	56.50	56.82
		64×64	73.72	36.16	54.50	54.11	55.97	62.54	59.33	60.93	50.17	69.71	60.17	61.37
		32×32	48.46	68.08	58.50	58.26	45.05	73.94	59.83	62.13	44.37	74.92	60.00	62.31
		16×16	61.43	57.33	59.33	60.37	64.85	58.63	61.67	62.04	63.83	56.35	60.00	61.09
		8×8	47.44	75.24	**61.67**	**62.04**	54.61	71.34	**63.17**	**65.34**	68.26	57.33	**62.67**	**63.75**
		*Mean*	*55.07*	*60.54*	*57.77*	*57.62*	*58.09*	*62.02*	*60.10*	*61.52*	*52.15*	*67.23*	*59.87*	*61.07*
		*Std Dev*	*12.31*	*15.05*	*3.06*	*3.81*	*9.66*	*12.02*	*2.52*	*2.94*	*14.01*	*9.93*	*2.20*	*2.59*
BBMAP-R	30	128×128	55.63	48.86	52.17	52.48	60.41	50.49	55.33	55.49	31.40	78.18	55.33	54.87
		64×64	38.91	65.15	52.30	52.02	42.66	65.15	54.17	53.82	48.46	69.38	59.17	58.97
		32×32	52.22	49.84	51.00	51.28	52.56	55.70	54.17	54.31	62.46	56.68	59.50	59.85
		16×16	36.18	80.78	59.00	58.19	69.97	47.23	58.33	59.09	50.85	70.68	61.00	61.30
		8×8	35.84	77.85	57.33	57.21	77.47	43.97	60.33	60.51	49.83	67.10	58.67	58.45
		*Mean*	*43.76*	*64.50*	*54.36*	*54.24*	*60.61*	*52.51*	*56.47*	*56.64*	*49.83*	*67.10*	*58.73*	*58.69*
		*Std Dev*	*9.44*	*15.03*	*3.56*	*3.21*	*13.77*	*8.29*	*2.75*	*2.99*	*11.12*	*7.76*	*2.09*	*2.39*
BBLL-R	30	128×128	62.80	43.97	53.17	52.18	18.43	92.83	56.50	54.57	32.08	80.78	57.00	56.75
		64×64	27.65	80.78	54.80	52.30	59.73	57.33	58.50	58.30	47.44	71.34	59.67	61.73
		32×32	83.96	22.48	52.50	51.69	37.88	82.08	60.50	62.64	66.55	57.34	**61.83**	**62.32**
		16×16	56.31	64.17	**60.33**	61.43	48.12	74.27	61.50	61.93	51.53	66.78	59.33	61.40
		8×8	39.25	79.48	59.83	**61.82**	62.45	60.91	**61.67**	**65.24**	63.14	57.65	60.83	62.00
		*Mean*	*53.99*	*58.18*	*56.13*	*55.88*	*45.32*	*73.48*	*59.73*	*60.54*	*52.15*	*66.78*	*59.73*	*60.84*
		*Std Dev*	*21.75*	*24.88*	*3.71*	*5.25*	*17.94*	*14.73*	*2.20*	*4.16*	*13.73*	*9.86*	*1.82*	*2.31*

The top performances of 10- and 30-fold cross-validation are shown in bold.

Another general comparison can be made with the cases of equal ROI and block sizes, for example when we use 64×64 block size in MIAS experiments. These cases are equivalent to conventional SRC, proposed by (35) and do not perform ensemble classification. Hence, these are ablation tests for the ensemble stage of our framework. The results indicate that our SLESA techniques outperform conventional SRC in both datasets. This is because block decomposition reduces the dimensionality of the images and enables the creation of multiple overcomplete dictionaries. An additional benefit is that we train multiple dictionaries on the same set of ROIs and fuse the residuals of multiple approximations to improve the classification accuracy.

**Figure 3:**
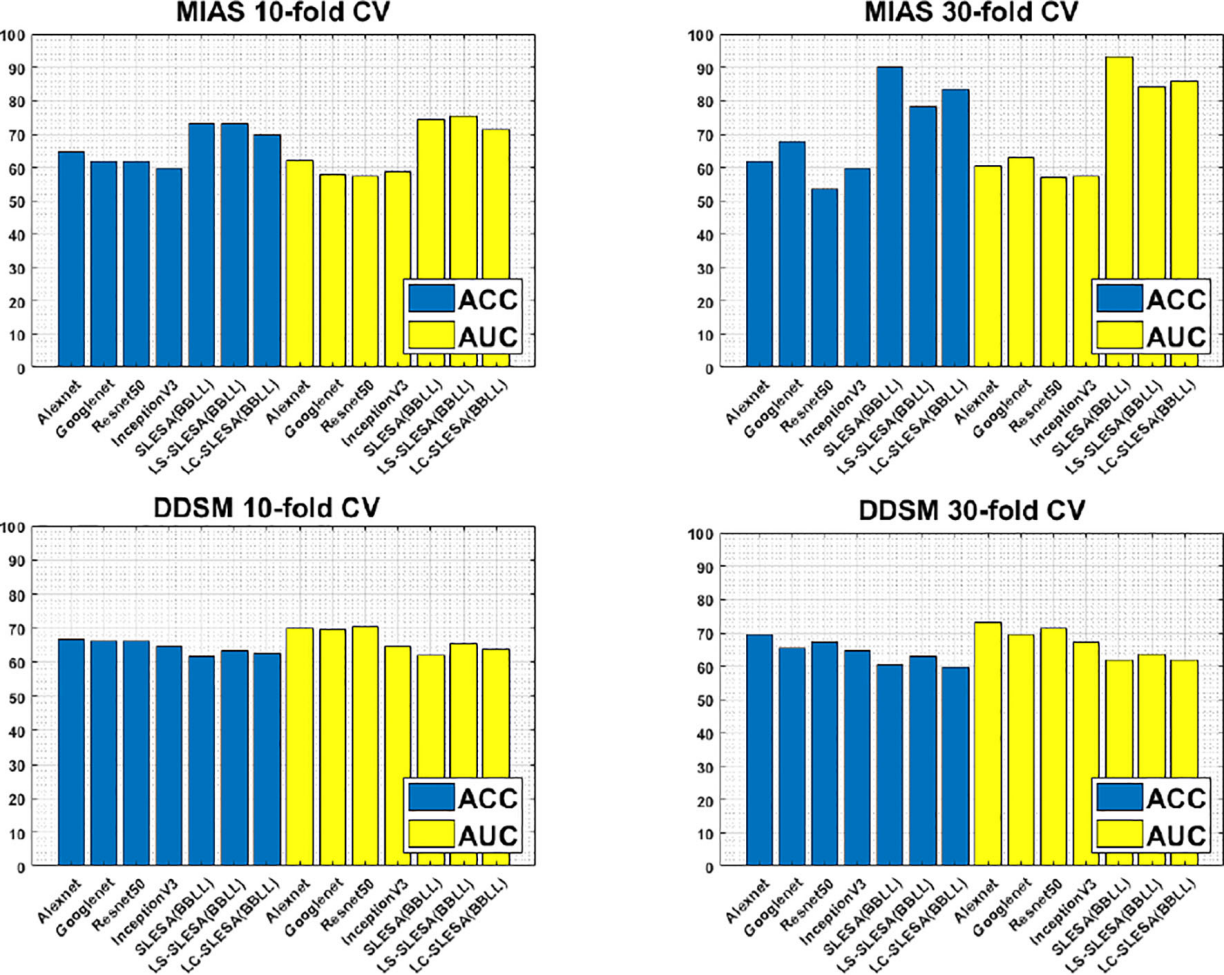
ACC performance comparisons on MIAS (top row) and DDSM (bottom row) datasets using 10- and 30-fold cross-validation.

Furthermore, **Figure 3** compares the ACC and AUC values of Alexnet, Googlenet, Resnet50 and InceptionV3 with SLESA, LS-SLESA and LC-SLESA. We observe that sparse approximations yield clearly better results on MIAS data, while CNNs with transfer learning are a bit more accurate on DDSM data.

We highlight the top AUC performances of CNNs and sparse methods per CV fold and dataset in **Table 5**. Our observations here are consistent with those we made in **Figure 3**. Our SLESA methods significantly outperform the best CNN performance on the MIAS dataset. On the DDSM dataset, the top CNN performances are slightly better than the SLESA counterparts in 10-fold CV, and the difference increases a bit in 30-fold CV. The size of the dataset may play a role in this difference, as neural networks learn best with large amounts of data. Additionally, the complexity of finding sparse solution in our sparse analysis methods increases as a larger amount of training samples are learned. Overall, the results indicate that sparse approximations produce good results on both datasets. In addition, they require fewer training data than CNNs, hence can produce better results than CNNs for smaller datasets.

**Table 5:** Top AUC performances of sparse analysis and deep learning methods on MIAS and DDSM datasets.

Dataset	k-Fold	Method	Block Size	TPR	TNR	ACC	AUC
	CV			(%)	(%)	(%)	(%)
MIAS	10	Alexnet	N/A	56.86	72.55	64.71	62.19
		SLESA	16 × 16	59.46	84.85	71.43	74.37
		LS-SLESA (BBLL-S)	8 × 8	59.46	81.82	70.00	75.35
		LC-SLESA (BBLL-S)	16 × 16	64.86	75.76	70.00	71.42
MIAS	30	Googlenet	N/A	66.67	68.63	67.65	63.04
		SLESA (BBLL-S)	8 × 8	96.77	82.76	90.00	93.10
		LS-SLESA (BBLL-S)	8 × 8	74.19	89.66	81.67	82.43
		LC-SLESA (BBLL-S)	16 × 16	96.77	68.97	83.33	88.43
DDSM	10	Resnet50	N/A	56.42	75.31	66.05	70.35
		SLESA (BBLL-R)	8 × 8	47.44	75.24	61.67	62.04
		LS-SLESA (BBLL-R)	8 × 8	54.61	71.34	63.17	65.34
		LC-SLESA (BBLL-R)	8 × 8	68.26	57.33	62.67	63.75
DDSM	30	Alexnet	N/A	72.64	66.55	69.59	73.04
		SLESA (BBLL-R)	8 × 8	39.25	79.48	59.83	61.82
		LS-SLESA (BBLL-R)	8 × 8	48.12	74.27	61.67	65.24
		LC-SLESA (BBLL-R)	32 × 32	66.55	57.34	61.83	62.32

**Figure 4:**
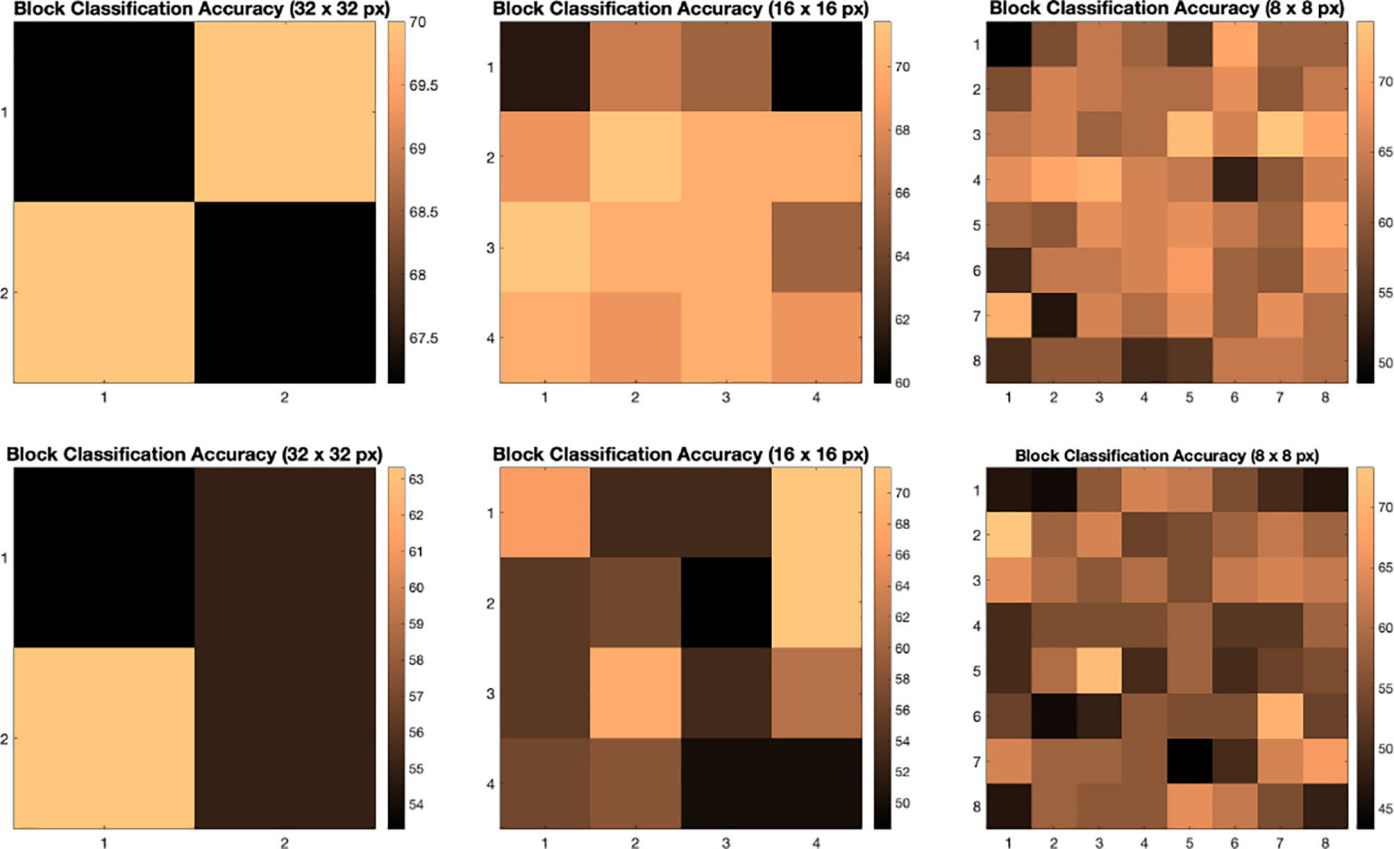
Classification accuracy by block for 32 **×** 32, 16 **×** 16, and 8 **×** 8 block experiments performed on the MIAS dataset for 10 fold CV (top row) and 30 fold CV (bottom row). The corresponding ensemble BBMAP-S and BBLL-S classification decision ACCs for 10-fold experiment examples are 70.00%, 70.00%, 70.00%, and 70.00%, 70.00%, 71.43% respectively for 32, 16, and 8 blocks. The corresponding ensemble BBMAP-S and BBLL-S classification decision ACCs for 30-fold experiment examples are 53.33%, 48.33%, 66.67%, and 80%, 90%, 90% respectively for 32, 16, and 8 blocks.

**Figure 5:**
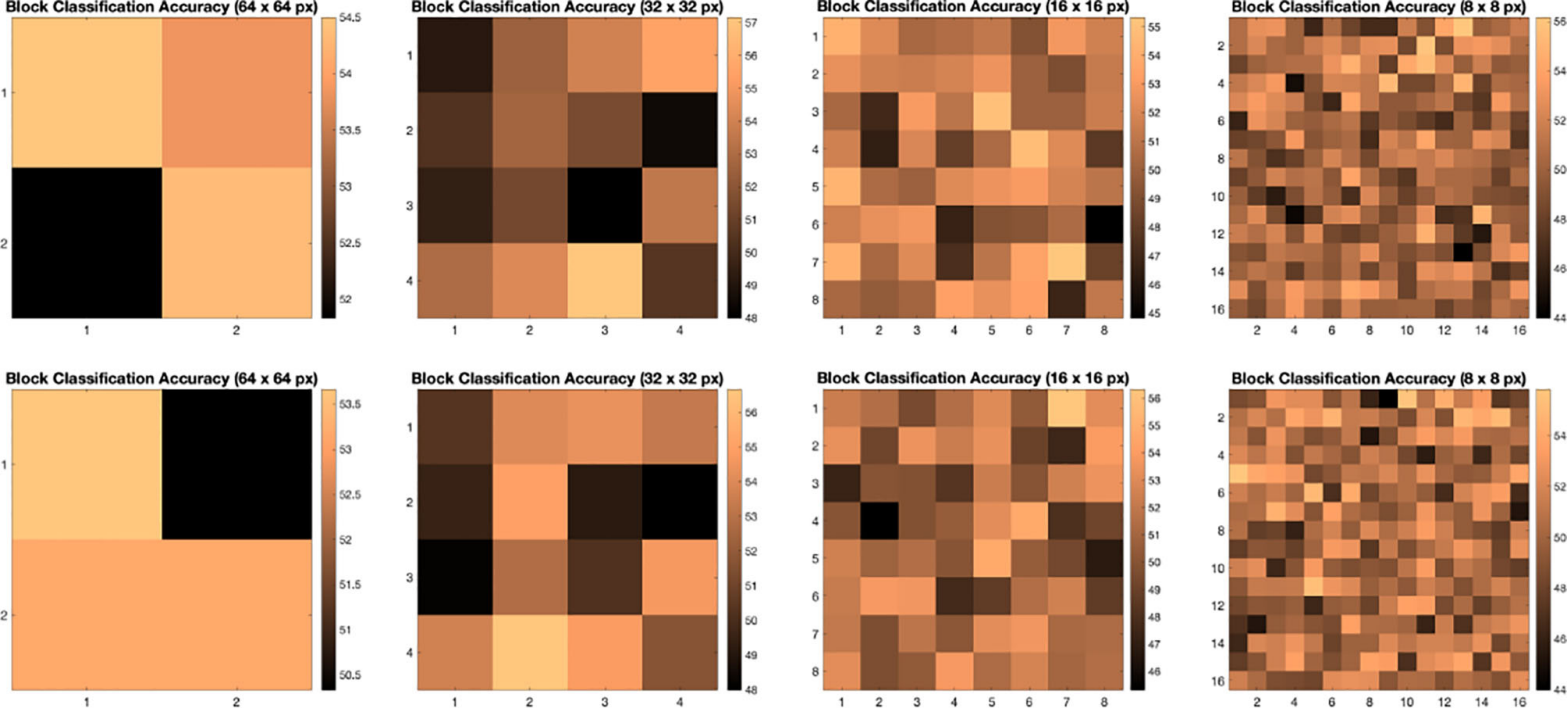
Classification accuracy by block for 64 **×** 64, 32 **×** 32, 16 **×** 16, and 8 **×** 8 block experiments performed on the DDSM dataset for 10 fold CV (top row) and 30 fold CV (bottom row). The corresponding ensemble BBMAP-R and BBLL-R classification decision ACCs for 10-fold experiment examples are 57.33%, 53.67%, 56.17%, 54.67%, and 59.50%, 59.67%, 60.17%, 57.33% respectively for 64, 32, 16, and 8 blocks. The corresponding ensemble BBMAP-R and BBLL-R classification decision ACCs for 30-fold experiment examples are 54.67%, 57.00%, 57.67%, 54.50% and 55.00%, 61.33%, 59.50%, 58.83% respectively for 64, 32, 16, 8 blocks.

We illustrate the effect of block localized learning on classification by performing block experiments on both datasets and comparing the classification rates per block. We include example block ACC experiment results in **Figures 4** and **5**. In MIAS block ACC experimentation we notice that top block ACC rates increase as the block size decreases, which confirms our expectation. A comparison between the top individual block ACCs and the ensemble BBLL rates reported in both **Figures 4**, **5** shows that BBLL is effectively combining block-based predictions to produce equivalent or improved ACC rates. In the block ACC experiments on DDSM (**Figure 5**), we observe consistent patterns of block ACC rates between 10-fold and 30-fold CV for all block sizes except for 64×64 px. While ensemble classification has its limitations, such as increased complexity in configuration and training, we see that ensembling reduces the variance and bias of classification.

**Figure 6:**
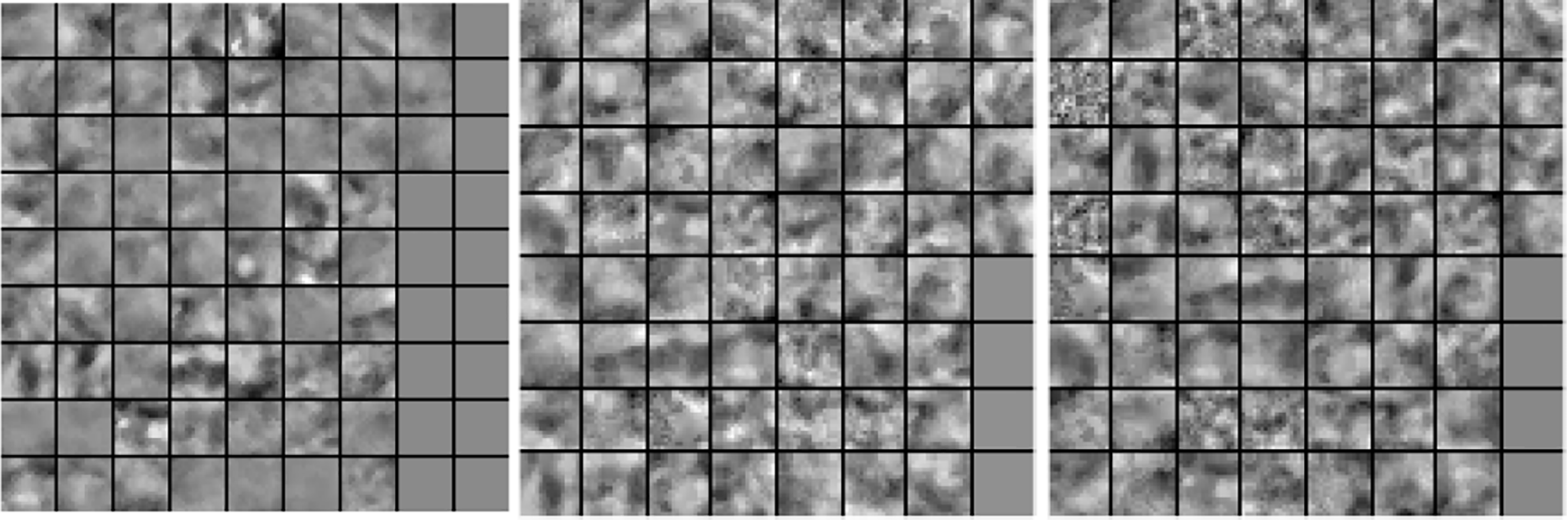
Dictionary comparison example for SLESA without dictionary learning (left), LS-SLESA (middle), and LC-SLESA (right).

In our next experiment, we explored the dictionaries learned by LS-SLESA and LC-SLESA in terms of visual pattern representation and inter-class separability. **Figure 6** displays examples of dictionaries produced by LS-SLESA and LC-SLESA based on 16×16 blocks from 64×64 ROIs of the MIAS database. We also display the training set for reference. These blocks correspond to one of the *D^j^* dictionaries defined in (3) and computed by (4) and (5). They were spatially localized –7th in lexicographical order out of a 4×4 grid. We see that the dictionary atoms correspond to basic structural patterns of the intensity distribution and texture of the masses.

**Figure 7:**
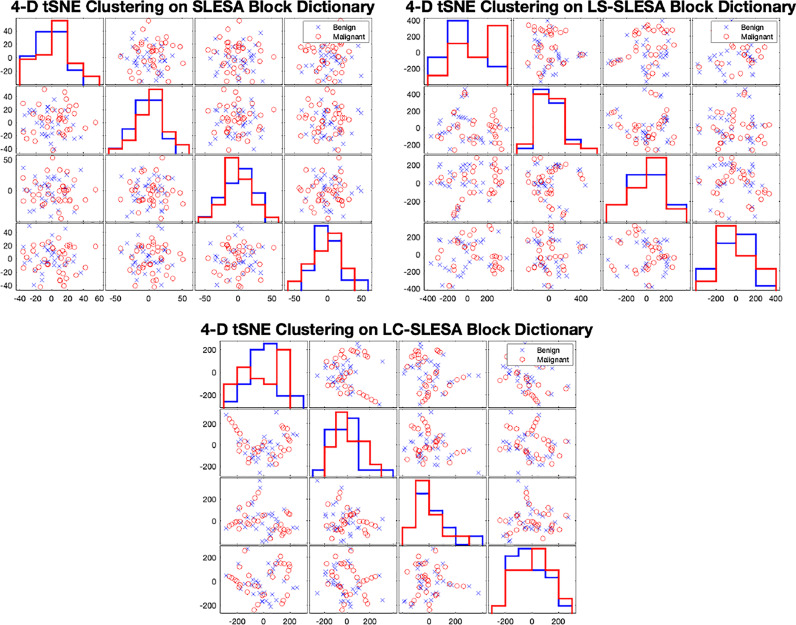
t-SNE clustering plots with 4-D embedding of block dictionaries produced by SLESA (top-left), LS-SLESA (top-right), and LC-SLESA (bottom). The greatest KL divergence for SLESA is 3.9353 produced by the first feature. The greatest KL divergence for LS-SLESA is 4.7651 produced by the third feature. The greatest KL divergence for LC-SLESA is 4.7252 produced by the first feature.

In **Figure 7** we display the 4-D t-SNE (40) clustering-based embeddings of dictionaries produced under the same conditions as **Figure 6** by LS-SLESA and LC-SLESA. This figure displays pair-wise feature scatterplots and single feature histograms grouped by the mass state. We include a t-SNE clustering plot of the training data without dictionary learning for comparison. We observe greater separation between class dictionaries when dictionary learning is applied to the training data. We also computed the symmetric Kullback Leibler (KL) divergence between the classes of benign and malignant samples in the embedded spaces to measure the level of inter-class separation. The greatest KL divergence of 4.7651 occurs in the third feature embedding of the LS-SLESA block dictionary and the second highest KL divergence, 4.7252, occurs in the first feature embedding of the LC-SLESA block dictionary. The observed separation constitutes the presence of similarities within class specific samples and further illustrates the benefit of dictionary learning on the training samples.

In both the MIAS and DDSM experiments we performed parameter optimization on the sparse techniques using grid search. In SLESA we used ∈ values of {0.001,0.01,0.1,0.5}. In LS-SLESA we added to the search, sparsity levels of {1,5,10,30,60}, and dictionary sizes of {300,500} atoms for DDSM. For the MIAS data, we used 60 atoms because of the small sample size. In LC-SLESA we added to the search, $\left(\sqrt{\alpha ,}\sqrt{\beta }\right)$ values of {(4*e*–4,2*e*–4), (4*e*–3,2*e*–3), (0.04,0.02), (0.4,0.2)}.

## 4 Conclusion

We introduced discriminative localized sparse representations to classify breast masses as benign or malignant using mammograms. LS-SLESA and LC-SLESA were designed to incorporate class-based discriminant information into the generative method of sparse representation using dictionary learning. We incorporated these approaches into a spatially localized ensemble learning methodology and extensively evaluated their classification performance. As we observed through our experimentation, these approaches produce sparse approximations that improve the classification accuracy and accomplish 93.1% area under the ROC using 30-fold cross-validation. Our results indicate that this methodology may be applicable for breast mass characterization in a breast cancer screening workflow.

## Data Availability Statement

The raw data supporting the conclusions of this article will be made available by the authors, without undue reservation.

## Ethics Statement

Ethical review and approval was not required for the study on human participants in accordance with the local legislation and institutional requirements. Written informed consent for participation was not required for this study in accordance with the national legislation and the institutional requirements.

## Author Contributions

SM designed and implemented the methods, wrote the manuscript, and performed experiments. KZ designed and implemented the methods, and performed experiments. CH implemented methods and performed experiments. All authors contributed to the article and approved the submitted version.

## Funding

This research was supported by the National Institute of General Medical Sciences of the National Institutes of Health (NIH) (award no.: SC3GM113754) and by the Army Research Office under grant no. W911NF2010095. We acknowledge the support by Delaware CTR-ACCEL (NIH U54GM104941) and the State of Delaware.

## Conflict of Interest

The authors declare that the research was conducted in the absence of any commercial or financial relationships that could be construed as a potential conflict of interest.

## Publisher’s Note

All claims expressed in this article are solely those of the authors and do not necessarily represent those of their affiliated organizations, or those of the publisher, the editors and the reviewers. Any product that may be evaluated in this article, or claim that may be made by its manufacturer, is not guaranteed or endorsed by the publisher.
